# The Rating of Charities

**Published:** 1888-10-13

**Authors:** 


### The Hospital

A Weekly Institutional Journal of

Science, Medicine, Nursing, and Philanthropy.

*For Hospitals, Asylums, Medical Practitioners, Students, Nurses, Families, Parishes, Congregations, and the Charitable Public.*

Vol. V. No. 107.] [October 13, 1888.

### THE RATING OF CHARITIES.

Hospital and Charity Managers, no doubt, like other people, are now returning to their work after the holidays imbued with a desire for energetic action and employment. No more suitable occasion could perhaps be selected for directing their attention to some of the more difficult problems presented by the management of the institutions for the efficiency of which they are responsible to the governors. It cannot be gainsaid that many metropolitan institutions find it very difficult to secure efficient and diligent governors as members of their committees. In the provinces the contrary is the case, because it is there held to be an honour to be associated with the management of the chief local charities. The effect of this non competition and indifference in London is conspicuous in the results achieved by metropolitan and provincial hospitals. In London a pound sterling goes little further if at all than half that sum in the provinces, where it is carefully expended under the active criticism of an energetic committee of governors. In such circumstances few London committeemen who are worthy of the name will be satisfied till they have ascertained the causes which have brought the expenditure to a sum per bed so large as to be astonishing when compared with towns of the size of Manchester, Birmingham, and Glasgow. We are going very carefully into the figures, and propose to publish the result of our investigations. Those managers who will follow our example must feel, with one of the most energetic and successful of hospital managers in London, "that the more time he spends in analysing and comparing the expenditure of metropolitan and provincial hospitals, the more he becomes convinced that it is impossible to find good cause why the cost per bed should be so much more in London." It is therefore of the greatest public importance that the committee of every London hospital should go into this question thoroughly and act upon the knowledge they may thus obtain. In the same way inquiry will show how much more thorough and successful has been the action of the provincial hospital managers in the matter of the rating of charities.

Our readers will no doubt have seen a correspondence in the *Times* during the past few weeks on the taxation of charities. That correspondence, though useful, is not calculated to immediately advance the question much, we fear, for reasons which will appear later. It is not generally known that all hospitals and kindred charities enjoyed exemption from rates until 1866. In Ireland and Scotland the practice of two centuries and a-half holds good, and the great charities in those countries are still exempt. Not only so, but in Ireland especially the hospitals are subsidised from the rates. The extent to which this system prevails will be apparent from the case of one of the county hospitals where the subsidy amounts to something like two-thirds of the expenditure. In Scotland the hospitals pay no rates, and in a recent case in the Law Courts Lord Shand laid it down that "the exemption extends to funds given to relieve physical privation or suffering arising from poverty." The justice, apart from the wisdom of compelling English hospitals alone to pay rates, does not appear, and the accident of the Mersey Docks case decision would seem to be shallow ground for enforcing so heavy a claim against charities which equity and practice had freed from such imposts for centuries. Still the opinion of successive Parliaments has been found to be against the entire exemption of hospitals from rates, and those managers who have recognised the inevitable and have come to terms with the overseers, aided by magisterial authority, have shown a wise discretion by saving their institutions from any but nominal payments for rates. In the provinces the latter practice has been pursued with universal success. In London, hospital managers have with few exceptions left the case in the hands of their solicitors, and have so been made to pay rates to an amount which has often crippled their resources.

We fully recognise that in times past too numerous exemptions have prevailed. These have led to great injustice, because in principle an exemption means that just in proportion to the relief from taxation given to any part of a community, some other portion of the same community is required to pay this taxation, in addition to the burdens that already fall upon it. The true and only principle of taxation is beneficial interest, and any attempt to go beyond this must launch statesmen into a sea of difficulties. Now charitable institutions are not places in which the subscribers have any beneficial interest. Such institutions are everywhere supported by a comparatively small portion of the population, and income so subscribed has already been taxed before it is given in charity, so that it is taxed twice over in the case of voluntary hospitals upon which rates are levied, whilst that part of a person's income which is not given in charity is only taxed once. Any principle of taxation, which includes hospitals and other institutions supported by voluntary contributions, is not, therefore, defensible in law or equity. Just in proportion, as the thousands of deserving persons, who otherwise would receive substantial relief from charitable institutions, fail to receive such relief on account of the pressure of rating, the rates are burdened; and this is a calamity of a most grievous kind. While we are strongly opposed to the different commercial exemptions which many advocate, believing them to be founded upon wrong principles altogether, we maintain that all that class of property, which consists of money devoted to charitable institutions, stands by itself, that there is no beneficial interest in it whatever, and that it is a beneficial interest only which ought to be taxed. We are, therefore, anxious, not only to prevent these institutions from being hampered with additional taxes, but we desire to relieve them, as far as possible, from the oppression of those taxes to which they are at present subjected.

Holding these views, we regret we cannot look with unmixed satisfaction upon the agitation with which the hospitals are at present threatened, with the object of securing them total exemption from all rates and taxes of every kind. If we believed there was any good reason to hope that the Bill it is proposed to introduce next session would receive Parliamentary sanction, we should joyfully welcome the movement. Unfortunately, anyone who has studied the history of this question in Parliament during the last twenty years, and who has taken the trouble to ascertain the views of the party leaders of all shades, must be convinced that it is desirable to let sleeping dogs lie. So far from securing further exemptions, there is good ground for fearing that the burden will ultimately be increased rather than diminished by any action of the kind at this time.

Those hospital managers who are wise, and who find the burden of the rates real and pressing, will do well to take the matter up with the overseers of their district, and not to rest content until they have obtained a fresh assessment based upon the principle of beneficial occupation only. Several of the best-managed hospitals have already taken this step, with the result that, as in the case of the Seamen's Hospital, Greenwich, for example, the rates have been reduced nearly seventy per cent.

To secure a similar result it must be remembered that if the occupation of the hospital can be shown to be technically beneficial it is not beneficial in the sense of property, because it is not a profitable occupation. Committees should maintain that they can only be lawfully rated to the extent of the legal beneficial occupation, *i. e.*, to the extent of the value of the ground before the hospital was erected, so as not to reduce the rateable value of the parish, with possibly a small sum beyond for the officers' quarters. No man would think of coming forward as tenant of a hospital and consent to carry it on year after year unless he were a philanthropist of a most extraordinary type. The view here stated is upheld by the cases of Staley *v.* the Overseers of Castletown, and Barter *v.* the Overseers of Salford, and is confirmed by the success which has attended the efforts of metropolitan and provincial hospital authorities who have put it to the test of practice. It rests with the committees concerned to look into these questions carefully for themselves. If they do so, we shall soon hear of the removal o many anomalies.

